# COS-7 and SVGp12 Cellular Models to Study JCPyV Replication and MicroRNA Expression after Infection with Archetypal and Rearranged-NCCR Viral Strains

**DOI:** 10.3390/v14092070

**Published:** 2022-09-17

**Authors:** Carla Prezioso, Sara Passerini, Dolores Limongi, Anna Teresa Palamara, Ugo Moens, Valeria Pietropaolo

**Affiliations:** 1 IRCSS San Raffaele Roma, Microbiology of Chronic Neuro-Degenerative Pathologies, 00163 Rome, Italy; 2 Department of Public Health and Infectious Diseases, Sapienza University of Rome, 00185 Rome, Italy; 3 IRCCS San Raffaele Roma, Telematic University, 00163 Rome, Italy; 4 Department of Infectious Diseases, Istituto Superiore di Sanità, 00161 Rome, Italy; 5 Laboratory Affiliated to Institute Pasteur Italia-Cenci Bolognetti Foundation, Department of Public Health and Infectious Diseases, Sapienza University of Rome, 00185 Rome, Italy; 6 Department of Medical Biology, Faculty of Health Sciences, University of Tromsø—The Arctic University of Norway, 9037 Tromsø, Norway

**Keywords:** COS-7, SVGp12, cellular models, JCPyV replication, miRNA expression, NCCR viral strains, exosomes

## Abstract

Since the non-coding control region (NCCR) and microRNA (miRNA) could represent two different and independent modalities of regulating JC polyomavirus (JCPyV) replication at the transcriptional and post-transcriptional levels, the interplay between JC viral load based on NCCR architecture and miRNA levels, following JCPyV infection with archetypal and rearranged (*rr*)-NCCR JCPyV variants, was explored in COS-7 and SVGp12 cells infected by different JCPyV strains. Specifically, the involvement of JCPyV miRNA in regulating viral replication was investigated for the archetypal CY strain—which is the transmissible form—and for the rearranged MAD-1 strain, which is the first isolated variant from patients with progressive multifocal leukoencephalopathy. The JCPyV DNA viral load was low in cells infected with CY compared with that in MAD-1-infected cells. Productive viral replication was observed in both cell lines. The expression of JCPyV miRNAs was observed from 3 days after viral infection in both cell types, and miR-J1-5p expression was inversely correlated with the JCPyV replication trend. The JCPyV miRNAs in the exosomes present in the supernatants produced by the infected cells could be carried into uninfected cells. Additional investigations of the expression of JCPyV miRNAs and their presence in exosomes are necessary to shed light on their regulatory role during viral reactivation.

## 1. Introduction

JC polyomavirus (JCPyV) is the etiological agent of progressive multifocal leukoencephalopathy (PML)—a fatal neurodegenerative disease characterized by a viral lytic infection of oligodendrocytes and astrocytes of the central nervous system [1,2]. JCPyV possesses a small double-stranded DNA genome, which can be divided into different regions. The early viral gene region (EVGR) encodes the large and small T antigens (LTAg and stAg), while the late viral gene region (LVGR) encodes the viral capsid proteins VP1, VP2, VP3, agnoprotein, and two mature microRNAs (miRNAs). Interposed between the EVGR and the LVGR is the non-coding control region (NCCR), which contains the origin of genome replication (ori) and the promoter/enhancer, with DNA-binding sites for several transcription factors (TFs) including, among others, a unique NF-κB site, C/EBPβ, NFAT4, Rad51, NF-1, and SP1 [3,4,5,6], able to mediate the host cell specificity [7], the timing of EVGR expression, the viral DNA replication, and the LVGR expression [8].

The NCCR is the most variable portion of the JCPyV genome, and sequence variation in the NCCR determines JCPyV tropism and its pathogenic effects [9,10,11]. Based on the NCCR structure, JCPyV can be referred to as having an archetypal or rearranged (*rr*) NCCR [12]. JCPyV with an archetypal non-pathogenic NCCR (CY strain) is considered to be the transmitted form, since it is found predominately in the kidneys or shed in the urine of healthy individuals, and it is rarely found in the brains of PML patients [13]. The archetypal CY NCCR is divided into six blocks, named A, B, C, D, E, and F [14]. While the ori and the F block are conserved among the different variants, the other blocks are highly variable. It is generally thought that the archetypal virus is the persistent form, whereas the rearranged virus is derived from the archetype during or after reactivation from persistence. The viral sequences associated with PML are known as PML-type and present significantly rearranged NCCRs [15]. The prototypical JCPyV NCCR sequence is designated by the MAD-1 variant, which was first isolated from the brain of a PML patient and is usually disease-associated [16]. The NCCR of MAD-1 is organized as follows: A, C, E, A, C, E, as 98 bp tandem repeats, followed by block F, and with blocks B and D deleted [13]. Such duplications and deletions generate more transcription factor binding sites (TFBSs) that could confer advantages to JCPyV [16]. A 98-base pair repeated in tandem is referred to as an enhancer element in the NCCR. The organization A, C, E, A, C, E contains duplicate TATA boxes, located in the A block, along with additional TFBSs [17,18]. The prevalence of these tandem repeats in the NCCR sequences of JCPyV isolated from PML patients suggests that these enhancer elements and the addition of TFBSs are critical for viral pathogenesis. Furthermore, the loss of the B block and D block can also result in increased viral gene expression. The deletions of both of these regions allow for additional TFBSs—such as YB-1/Purα and NF-1—to bind and to facilitate enhanced viral gene expression [19,20,21,22]. TFBS—such as Spi-B transcription factor (Spi-B), nuclear factor of activated T cells 4 (NFAT4), and subtypes of the nuclear factor 1 (NF-1) family—are also important in early gene transcription, and may play a role in cellular tropism [20]. Specifically, the archetypal NCCR has fewer binding sites for Spi-B, resulting in less efficient viral gene transcription when compared to the *rr*-NCCRs [23,24,25]. Rearrangements in the NCCR appear to emerge when JCPyV replicates in immunocompromised patients. In vitro studies with isolates of JCPyV containing *rr*-NCCRs from HIV patients with PML replicated better than archetypal JCPyV, suggesting that rearrangements in the NCCR contribute to the disease’s progression [26,27].

Moreover, in vitro studies support the observations that *rr*-NCCRs confer a higher replicative activity for a defect in the immune system functions in vivo [28]. Hence, NCCR rearrangements arising in immunocompromised patients could be considered to be not only a marker of viral replication, but also a virulence determinant of activated EVGR expression and increased replication capacity causing disease. It is not well-established whether JCPyV infection is a true latent infection characterized by the absence of gene expression, or is a persistent infection accompanied by a low level of active replication.

In addition to the NCCR, another layer regulating EVGR expression has been described at the post-transcriptional level, mediated by miRNAs encoded in the distal LVGR [29,30,31]. Similar to BK polyomavirus (BKPyV) [32], the JCPyV miRNAs JC-miRNA-3p and -5p, are short, mature, noncoding RNAs that are encoded by the late transcript and target the early mRNAs, such as LTAg mRNA [31]. One of the major functions of LTAg is driving DNA replication of the viral genome by binding directly to the ori, through its intrinsic helicase/ATPase activity, and by recruiting cellular replication factors [33,34]. LTAg is also responsible for the transcriptional switch from early to late, by directly activating late gene expression and downregulating its early promoter [35,36,37]. Since LTAg is responsible for initiating viral DNA replication [38], it is reasonable to speculate that, by downregulating LTAg expression, miRNAs may play a role in limiting viral replication, helping the virus to escape elimination by the immune system [31,39]. Consequently, viral miRNAs and their genetic variability may also play roles in the induction of JCPyV reactivation, the success of persistence or replication, and the development of diseases [31,39]. The regulation of miRNAs’ expression is the subject of ongoing studies, and may involve sequences close to the miRNA gene as well as to the NCCR. As has been previously proposed for BKPyV, miRNA is able to regulate EVGR expression late in the infection, because it is expressed from the late strand and is complementary to early mRNAs [40]. Subsequently, evidence has shown that miRNAs can regulate early mRNA expression prior to genome replication. This regulation is accomplished through the control of early gene and miRNA expression influenced by the delicate balance of elements within the NCCR.

Since the NCCR and miRNA could represent two different and independent modalities of regulating JCPyV replication at the transcriptional and post-transcriptional levels, respectively, in this study, the interplay between JC viral load based on the NCCR’s structural architecture and miRNA levels, following JCPyV infection with archetypal CY and *rr*-NCCR-JCPyV MAD-1 variants, was explored. Moreover, increasing evidence has shown that exosomes may be exploited by human viruses such as HIV, HSV, and EBV, to provide additional routes for the transmission of viral infections. This exosome-mediated spread evades immune recognition and exerts inflammatory effects on cells, thereby facilitating persistent infections in the host [41,42,43]. On this basis, whether the JCPyV miRNAs in the exosomes produced by the infected cells might be carried into uninfected cells was also verified.

Our results could help to clarify aspects regarding viral persistence in human hosts, and could contribute to defining the role of miRNAs as viral factors involved in JCPyV’s persistence and reactivation mechanisms.

## 2. Materials and Methods

### 2.1. Cell Cultures, Transfection, and Infection

Two different cell lines—COS-7, an African green monkey cell line transformed with an origin-defective mutant of SV40 [44]; and SVGp12, a cell line established by immortalization of human fetal glial cells with an origin-defective mutant of SV40 [45]—were obtained from the American Type Culture Collection (ATCC, Manassas, VA, USA). The cells were cultured in Dulbecco’s modified Eagle medium (DMEM) and Eagle’s minimal essential medium (EMEM), supplemented with 100 U of penicillin and 100 μL of streptomycin per mL, respectively (Sigma-Aldrich S.r.l., Milano, Italia), and fetal bovine serum (FBS) (10%). The cells were incubated at 37 °C in the presence of 5% CO_2_ and propagated at a ratio of 1:4 or 1:8. The cells were then plated at a density of 7.5 × 10^4^ and grown for 24 h in complete growth medium to reach 50–70% confluence on the day of transfection. The cells were then transfected with 1.5 μg of JCPyV CY strain DNA and with 1.5 μg of JCPyV MAD-1 strain DNA, following the specifications of the Xfect TM Transfection Reagent kit (Clontech Laboratories, Inc., Mountain View, CA, USA). The cells were incubated at 37 °C for 4 h with the transfection mixture. After two washes with phosphate-buffered saline (PBS), the cells were incubated with complete culture medium for the time-course experiment. After two days of incubation, the cells were transferred to a 34 mL flask, and then continuously cultured in the maintenance medium with transfer at a split ratio of 1:4 every 3–4 days. Supernatants and cellular fractions were harvested and stored until 35 days post-transfection (d.p.t.). Supernatants harvested from previous transfection experiments with the JCPyV CY and MAD-1 strains’ DNA were subjected to 6 cycles of freezing and thawing, followed by centrifugation at 2000 rpm for 5 min.

In order to avoid different viral DNA copies being used in the infection experiments, the resulting clarified supernatants were analyzed by qPCR, and supernatants containing virions corresponding to 1 × 10^5^ copies per milliliter (copies/mL) were selected to infect freshly seeded COS-7 and SVGp12 cells. After adsorption for 2 h, the cells were washed 3 times with PBS and incubated with fresh medium for 4 days, before being transferred into 90 mm diameter dishes. Subsequently, the cells were continuously cultured in the maintenance medium. Cells and supernatants were collected once a week until 35 days post-infection (d.p.i.).

### 2.2. JCPyV DNA Extraction from COS-7 and SVGp12 Cells

Total DNA was extracted from 1 × 10^6^ COS-7 and SVGp12 cells using a QIAamp^®^ DNA Mini Kit (QIAGEN S.p.A., Milan, Italy), following the instructions provided by the manufacturer. Once extracted, the DNA was stored at −20 °C until use. Supernatants from the COS-7 and SVGp12 cells were initially subjected to six cycles of freezing and thawing, and then centrifuged at 2000 rpm for 10 min. The resulting clarified supernatant was used directly in molecular biology assays.

### 2.3. Quantitative Real-Time PCR (qPCR) for JCPyV LTAg DNA

The extracted DNA was analyzed using a TaqMan real-time PCR (qPCR) to detect and quantify the JCPyV *LTAg* gene with a 7300 Real-Time PCR System (Applied Biosystems, Waltham, MA, USA), following a previously published protocol [46]. Each sample was analyzed in triplicate, and the viral loads were given as the mean of at least three positive reactions. Standard precautions designed to prevent contamination were followed, and a negative control was included in each run. Viral DNA was quantified using a standard curve consisting of serial dilutions of a plasmid containing the entire JCPyV genome with a known titer (range, 10^5^ copies/mL–10^2^ copies/mL). The amount of cellular DNA was quantified simultaneously using SYBR Green PCR for the housekeeping *β-globin* gene [47] and used to normalize the JCPyV DNA. The data were expressed as copies of viral DNA per cell based on DNA content (copies/cell) for the COS-7 cells, and as copies of viral DNA per milliliter (copies/mL) for the supernatants.

### 2.4. Western Blot (WB) Analysis to Study VP1 Expression

Supernatants harvested from previous transfection experiments with JCPyV CY and MAD-1 DNA strains, and containing virions corresponding to 1 × 10^5^ copies per milliliter (copies/mL), were used to infect freshly seeded COS-7 and SVGp12 cells. To confirm that expression of the JCPyV late genes occurred in the COS-7 and SVGp12 cell lines, JCPyV VP1 expression was regularly monitored by Western blot (WB) analysis once per week until 35 d.p.i. The concentrations of proteins in each supernatant were determined using the BCA protein assay kit (Pierce BCA Protein Assay Kit, Thermo Scientific, Waltham, MA, USA). Equal amounts of proteins from supernatants and whole-cell protein extracts were separated by sodium dodecyl sulfate–polyacrylamide gel electrophoresis (SDS–PAGE), transferred to polyvinylidene fluoride (PVDF) membranes (Bio-Rad, Hercules, CA, USA), and blocked with 5% non-fat dry milk (NFDM, Bio-Rad). The membranes were incubated with the murine monoclonal anti-human polyomavirus JCV capsid protein VP1 antibody (1:400 dilution; ab34756 Ab-cam) and with the anti-mouse HRP-conjugated antibody (Bio-Rad). To normalize the total protein contents in COS-7 and SVGp12 cells, blots were probed for glyceraldehyde-3-phosphate dehydrogenase (GAPDH) using the anti-GAPDH antibody (1:8000 dilution; ab9485 Abcam, Cambridge, UK). The signals were detected with enhanced chemiluminescence reagents (GE, Healthcare, Chicago, IL, USA). Band intensities were quantified by densitometric analysis using the ImageJ software [48].

### 2.5. Viral miRNAs Detection and Quantification

Total RNA, including miRNA, was extracted from cell pellets using the miRNeasy kit (QIAGEN) according to the manufacturer’s instructions. RNA quality and quantity were assessed using A230/A260 ratios. MiRNA expression was analyzed and quantified with the specific JCPyV miR-J1-5p quantitative TaqMan real-time PCR (Applied Biosystems 7300), as described previously [49]. Each reaction was carried out in triplicate with 15 ng of RNA and including negative controls. Synthetized oligonucleotides were used as standards (dilution range: 10^1^–10^6^ copies). The lower limit of detection was 10 copies of viral miRNA per ng of RNA. Human let7 miRNA was used as a control for endogenous miRNA expression in each assay [50]. One hundred nanograms of the JCPyV DNA of selected samples was PCR-amplified employing primers specific to the miRNA-expressing regions, purified using the PCR Purification Kit (QIAGEN, Milan, Italy), and sequenced at a dedicated facility (Bio-Fab Research, Rome, Italy). The obtained sequences were aligned and analyzed using the ClustalW algorithm in BioEdit 7.2 (Tom Hall of Ibis Therapeutics, Carlsbad, CA, USA), applying THE default parameters.

### 2.6. Exosome Extraction from Cell Cultures and Addition of JCPyV miRNA-Containing Exosomes to Uninfected Cell Lines

The exosomes were isolated starting from 250 μL of cell supernatant collected by prior centrifugation at 14,000× *g* for 20 min, using the exosome-specific extraction kit (Norgen, Thorold, ON, Canada) according to the manufacturer’s protocols. Extracted miRNAs were retro-transcribed and quantified by JCPyV-miRNA qPCR, as described above.

To determine whether the JCPyV miRNA present in exosomes could be transported into the cells, the exosomes obtained from a cell-free supernatant of COS-7/SVGp12-infected cells and pretreated with RNAse to degrade the miRNAs not protected inside the exosomes, containing 500 copies of the JCPyV miRNA, were incubated with the uninfected COS-7 and SVGp12 cells at 37 °C for 2 h. Exosomes recovered from the cell-free supernatants of uninfected COS-7 and SVGp12 cells were used as controls. After cell treatment with trypsin and a washing step to remove residual viral particles and exosomes adsorbed to the cells, the treated cells were incubated at 37 °C for 2 h. Then, the cells were collected 24 h post-infection (h.p.i.). Viral DNA and miRNAs were extracted and quantified as described above.

### 2.7. Statistical Tests

The data were analyzed using Student’s *t*-test; *p*-values less than 0.05 were considered statistically significant.

## 3. Results

### 3.1. JCPyV Replication Rates in the COS-7 and SVGp12 Cell Lines

Intracellular viral DNA was extracted from 1 × 10^6^ COS-7 and SVGp12 cell lines at different cell sampling times (from 3 d.p.i. to 35 d.p.i.) and quantified by qPCR. Results obtained from three independent experiments confirmed that JCPyV DNA efficiently replicated in both cell lines, showing a progressive increase in viral DNA content.

**Figure 1:**
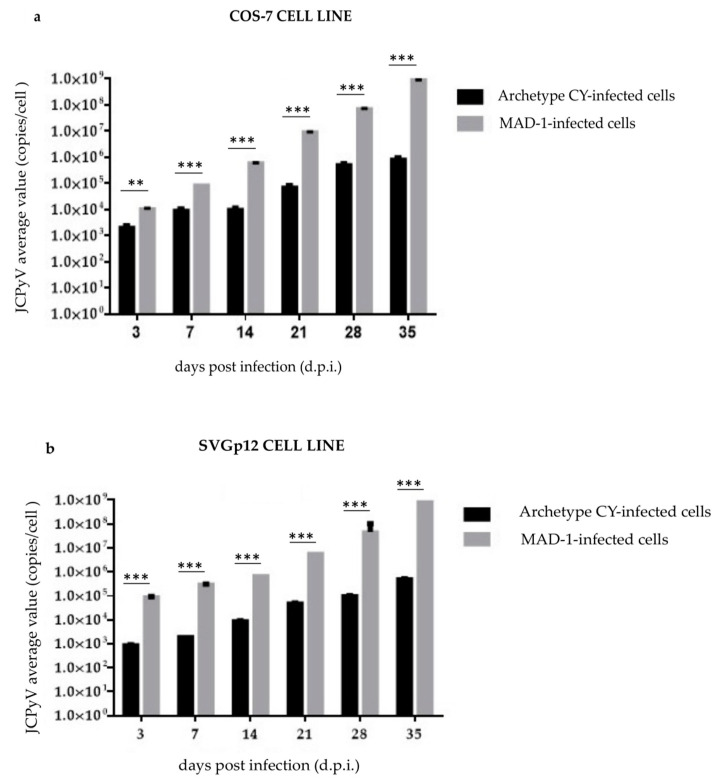
JCPyV replication in the CY- and MAD-1-infected COS-7 and SVGp12 cell lines: (**a**) Archetypal CY and MAD-1 strains compared to address the role of the NCCR in modulating viral replication into the COS-7 cell line at selected sampling times after infection, from 3 d.p.i until 35 d.p.i.; JCPyV DNA was quantified by qPCR. Data are expressed as the mean of three independent experiments, and error bars indicate standard deviations; ns: not significant; ** *p* < 0.01; *** *p* < 0.001. JCPyV-loads rapidly increased in MAD-1-infected cells, but only slowly in archetypal-CY-infected cultures. (**b**) Archetypal CY and MAD-1 strains compared to address the role of the NCCR in modulating viral replication into the SVGp12 cell line at selected sampling times after infection, from 3 d.p.i until 35 d.p.i.; JCPyV DNA was quantified by qPCR. Data are expressed as the mean of three independent experiments, and error bars indicate standard deviations. ns: not significant; *** *p* < 0.001. JCPyV-loads rapidly increased in MAD-1-infected SVGp12 cells, but only slowly in archetypal-CY-infected cultures.

Archetypal CY and *rr*-NCCR MAD-1 strains were compared to address the role of the NCCR in modulating viral replication. Accordingly, the DNA of these strains was transfected into COS-7 and SVGp12 cells, and infectious supernatants were prepared. Following infection, JCPyV-loads significantly increased in MAD-1-infected cells, but only slowly in archetypal-CY-infected cultures (*p* < 0.05) (Figure 1a,b).

Specifically, in the archetypal-CY-infected cells, viral DNA increased from 2 × 10^3^ copies/cell (3 d.p.i.) to 5 × 10^5^ copies/cell (28 d.p.i.) in COS-7 cells, and from 9 × 10^2^ copies/cell (3 d.p.i.) to 1 × 10^5^ copies/cell (28 d.p.i.) in SVGp12 cells (Table S1).

In parallel, JCPyV replication, evaluated by measuring viral DNA in the supernatant harvested at the same cell sampling times (from 3 d.p.i. to 28 d.p.i.), showed that the trend of the JCPyV load was essentially the same as that observed in the COS-7 and SVGp12 cells infected with the CY viral strain. The viral load inside the cells (Figure 1) and in the supernatants continuously increased up to 35 d.p.i (8 × 10^5^ copies/cell (35 d.p.i.) in COS-7 cells; 5 × 10^5^ copies/cell (35 d.p.i.) in SVGp12 cells) (Table S1). Results obtained from the analysis of JCPyV DNA replication rates in both cell lines infected by the MAD-1 strain showed JCPyV-loads that rapidly increased in the infected cells. The increase in the amount of viral DNA ranged from 1 × 10^4^ copies/cell (3 d.p.i.) to 9 × 10^8^ copies/cell (35 d.p.i.) in COS-7 cells, and from 9 × 10^4^ copies/cell (3 d.p.i.) to 8.5 × 10^8^ copies/cell (35 d.p.i.) in SVGp12 cells (Table S1). In parallel, JCPyV replication, evaluated by measuring viral DNA in the supernatant harvested at the same cell sampling times (from 3 d.p.i. to 35 d.p.i.), showed that the trend of the JCPyV load was the same as that observed in the MAD-1-infected COS-7 and SVGp12 cells. In all experiments, the uninfected COS-7 and SVGp12 cells were negative for the viral DNA replication.

### 3.2. miR-J1-5p Expression in the COS-7 and SVGp12 Cell Lines

To address the role of the NCCR in modulating the expression of JCPyV-encoded miRNAs, the archetype (CY strain) and *rr*-NCCR-JCPyV (MAD-1 strain) were transfected into COS-7 and SVGp12 cells, and infectious supernatants were prepared. Following infection, although the JCPyV load increased rapidly in MAD-1-infected cells and only slowly in CY-NCCR-JCPyV-infected cultures (Figure 1a,b), a progressive decrease in miR-J1-5p expression was observed over the course of experiments (Table S2).

Specifically, at 3 d.p.i., the mean expression level of miR-J1-5p in CY-COS-7-infected cells was 900 copies/ng of RNA (Table S2). After 7 d.p.i., the results of qPCR revealed a decrease in the mean miR-J1-5p value from 900 copies/ng to 800 copies/ng (Table S2). An additional decrease was observed in the subsequent sampling points (14–35 d.p.i.), from 700 (14 d.p.i.) to 540 copies/ng (35 d.p.i) (Table S2). In CY-SVGp12-infected cells at 3 d.p.i., the mean expression level of miR-J1-5p was 950 copies/ng of RNA (Table S2), and from 7 to 35 d.p.i. a further decrease was observed in the subsequent sampling points (Table S2).

**Figure 2:**
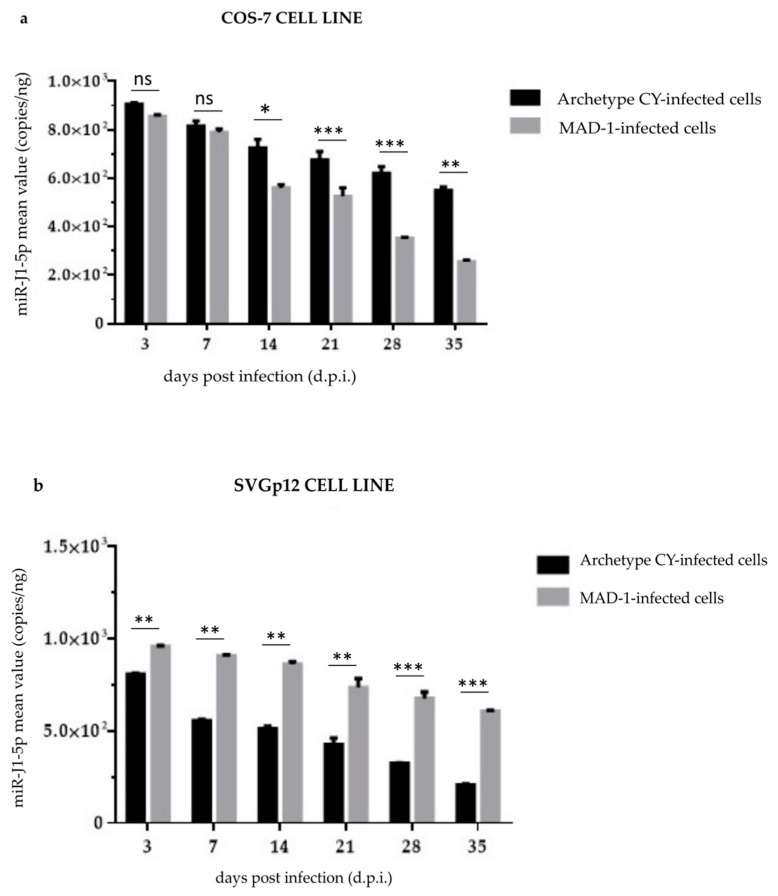
miR-J1-5p expression in the CY- and MAD-1-infected COS-7 and SVGp12 cell lines: (**a**) Archetypal CY and MAD-1 strains compared to address the role of the NCCR in modulating miR-J1-5p expression in the COS-7 cell line at selected sampling times after infection, from 3 d.p.i until 35 d.p.i.; JCPyV miR-J1-5p expression was quantified by quantitative TaqMan real-time PCR. Data are expressed as the mean of three independent experiments, and error bars indicate standard deviations; ns: not significant; * *p* < 0.05; ** *p* < 0.01; *** *p* < 0.001. The miRNA levels observed in the archetypal-CY-infected cells were lower than those observed in the MAD-1-infected cultures. (**b**) Archetypal CY and MAD-1 strains compared to address the role of the NCCR in modulating miR-J1-5p expression in the SVGp12 cell line at selected sampling times after infection, from 3 d.p.i until 35 d.p.i.; JCPyV miR-J1-5p expression was quantified by quantitative TaqMan real-time PCR. Data are expressed as the mean of three independent experiments, and error bars indicate standard deviations; ns: not significant; ** *p* < 0.01; *** *p* < 0.001. The miRNA levels observed in the archetypal-CY-infected cells were lower than those observed in the MAD-1-infected cultures.

Throughout the study (3 d.p.i. to 35 d.p.i.), the detection of miR-J1-5p was also performed on cells infected with the MAD-1 strain. At 3 d.p.i., in MAD-1-COS-7-infected cells, the mean miR-J1-5p value was 850 copies/ng of RNA; from 7 d.p.i. to 35 d.p.i., the qPCR showed a reduction in the mean miR-J1-5p value, from 780 copies/ng of RNA at 7 d.p.i. to 250 copies/ng of RNA 35 d.p.i. (Table S2). In MAD-1-SVGp12-infected cells, the mean miR-J1-5p value was 800 copies/ng of RNA at 3 d.p.i.; from 7 d.p.i. to 35 d.p.i., the qPCR showed a reduction in the mean miR-J1-5p value, from 550 copies/ng of RNA at 7 d.p.i. to 200 copies/ng of RNA at 35 d.p.i. (Table S2). A statistical analysis of miR-J1-5p expression in the COS-7 and SVGp12 cell lines evidenced that the miRNA levels detected in the MAD-1-infected cells were significantly lower than those observed in the archetype-CY-infected cultures (*p* < 0.05) after 14 d.p.i. in the COS-7 cells, and throughout the study in the SVGp12 cells (*p* < 0.05) (Figure 2a,b). In all experiments, the uninfected COS-7 and SVGp12 cells were negative for the miRNA expression. The JCPyV miRNA sequence variability was also investigated, and the miRNA-encoding region found in all of the JCPyV-DNA-positive cells was sequenced. No mutations were observed in any of the analyzed miR-J1-5p-positive samples.

### 3.3. Evaluation of JCPyV miRNA Expression in the Exosomes, and Study of Replication Following Post-Infection Addition of JCPyV-miRNA-Containing Exosomes to Uninfected Cell Lines

**Figure 3:**
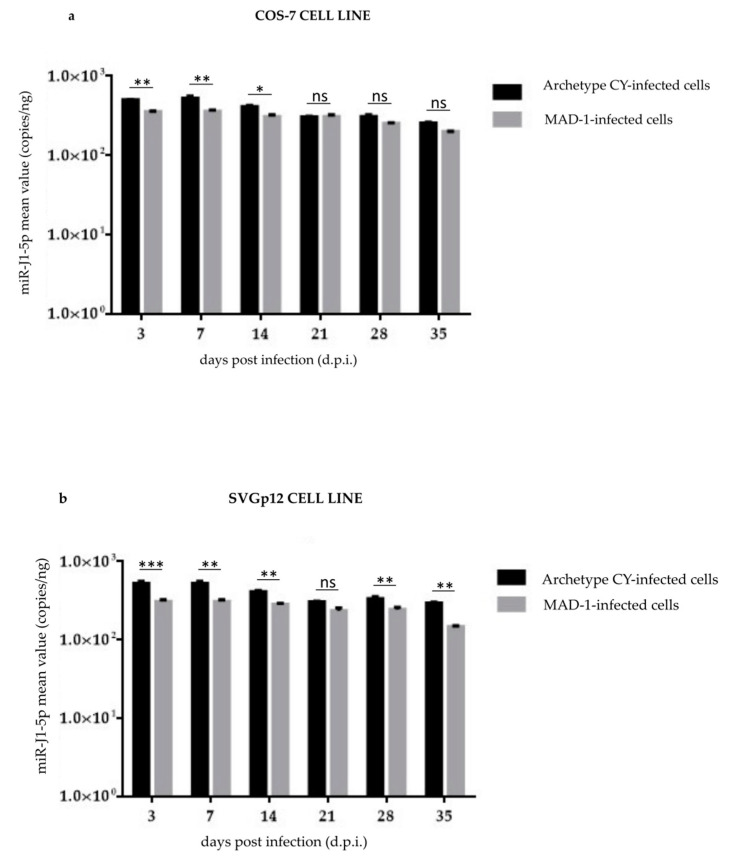
Expression of miRNA levels observed in exosomes generated during archetypal and MAD-1 JCPyV infection of COS-7 and SVGp12 cell lines: (**a**) JCPyV miR-J1-5p expression was quantified by quantitative TaqMan real-time PCR at selected sampling times after infection, from 3 d.p.i until 35 d.p.i.; data are expressed as the mean of three independent experiments, and error bars indicate standard deviations; ns: not significant; * *p* < 0.05; ** *p* < 0.01. The results showed that JCPyV miRNA was present in the exosomes starting from 3 d.p.i. during archetypal and MAD-1 JCPyV infection of the COS-7 cell line. A higher expression of miRNA levels was observed in exosomes generated during archetypal JCPyV infection than was observed for the *rr*-NCCR. (**b**) JCPyV miR-J1-5p expression was quantified by quantitative TaqMan real-time PCR at selected sampling times after infection, from 3 d.p.i until 35 d.p.i;. data are expressed as the mean of three independent experiments, and error bars indicate standard deviations; ns: not significant; ** *p* < 0.01; *** *p* < 0.001. The results showed that JCPyV miRNA was present in the exosomes starting from 3 d.p.i. during archetypal and MAD-1 JCPyV infection of the SVGp12 cell line. A higher expression of miRNA levels was observed in exosomes generated during infection with the CY strain than was observed for the MAD-1 strain.

To investigate whether the JCPyV miRNAs were expressed in the exosomes produced by COS-7 and SVGp12 cell-free supernatants, purified exosomes that tested positive for the tetraspanin CD63 protein were analyzed. The results showed that JCPyV miRNA was present in the exosomes starting from 3 d.p.i. A higher expression of miRNA levels was observed in exosomes generated during archetypal (CY) JCPyV infection than was observed for the JCPyV MAD-1 infection (Figure 3a,b). In the COS-7 cell line, the difference was considered statistically significant from 3 to 14 d.p.i. (*p* < 0.05), whereas in the SVGp12 cell culture, the difference was statistically significant at all analyzed times, except at 21 d.p.i. Detailed results are reported in Table S3.

For the first time, the replication of archetypal and MAD-1 JCPyV strains, along with viral miRNA expression in the exosomes within COS-7 and SVGp12 cell supernatants, was investigated at an early time point of infection (24 h.p.i.). Our results confirm the presence of replication and viral production in COS-7 (2.5 × 10^3^ copies/cells) and SVGp12 cells (5.5 × 10^3^ copies/cells) infected with the MAD-1 strain, along with the lack of viral replication in SVGp12 cells infected with the archetypal JC viral strain. A very low replication was detected in COS-7 cells (2.5 × 10^1^ copies/cell) infected with the CY strain.

JCPyV miRNA expression was consistently detected in well-characterized exosomes (CD63-positive) present at a high concentration in supernatants obtained from CY-infected cells, with a mean miR-J1-5p value of 400 copies/ng of RNA.

**Figure 4:**
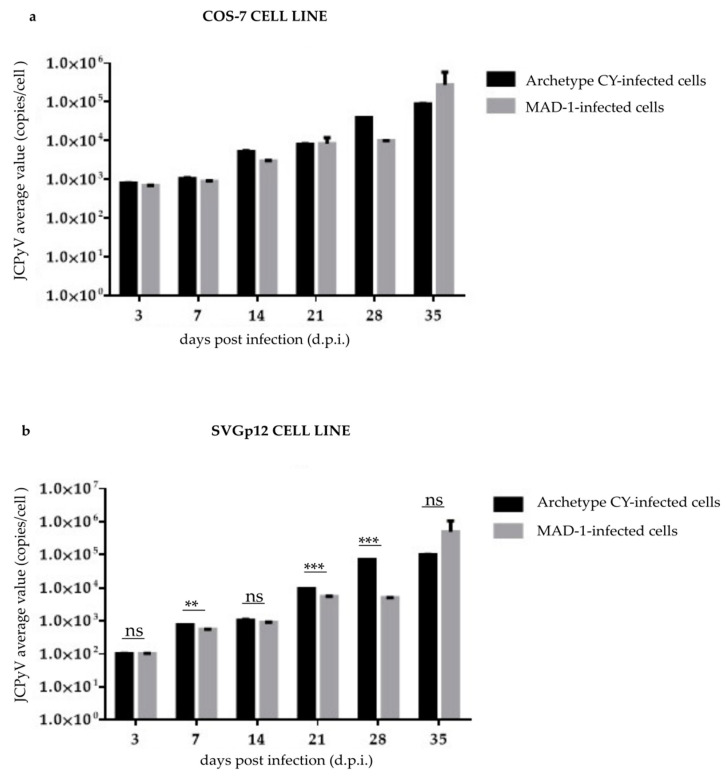
JCPyV replication in the CY- and MAD-1-infected COS-7 and SVGp12 cell lines after the addition of JCPyV-miRNA-5p exosome preparations to uninfected cells: (**a**) Viral replication into the COS-7 cell line at selected sampling times after infection, from 3 d.p.i until 35 d.p.i., was quantified by qPCR. Data are expressed as the mean of three independent experiments, and error bars indicate standard deviations; ns: not significant. (**b**) Viral replication into the SVGp12 cell line at selected sampling times after infection, from 3 d.p.i until 35 d.p.i., was quantified by qPCR. Data are expressed as the mean of three independent experiments, and error bars indicate standard deviations; ns: not significant; ** *p* < 0.01; *** *p* < 0.001.

To test whether the JCPyV miRNAs in the exosomes present in the supernatants, produced by the infected cells were able to downregulate the replication of different JCPyV strains, JCPyV-miRNA-5p-loaded exosome preparations were added to the cells prior to infection. Comparing the data obtained from replication in the absence of exosomes (Table S1) with those obtained from the replication in the presence of exosomes (Table S4), a degree of inhibition was observed—especially in the replication of the JCPyV-MAD-1 strain (Figure 4a,b). Detailed results are reported in Table 1. Specifically, the replication of CY in COS-7 cells was inhibited by 50–89% when the cells had been exposed to exosomes from infected cells compared to non-exposed cells. Pretreatment of COS-7 cells with exosomes from infected cells almost completely (≥99%) abolished MAD-1 replication. Similarly, comparing JCPyV replication in SVG12p cells not treated with exosomes with cells exposed to exosomes from JCPyV-infected cells showed 30–89% inhibition of CY replication and 99.9% or more inhibition of MAD-1 replication at the different time points tested. The replication of the JCPyV-MAD-1 strain was significantly reduced over the course of the experiment, whereas a significant—albeit less marked—effect was observed in both cell lines infected with archetypal CY JCPyV (*p* < 0.05) (Figure 4a,b).

### 3.4. VP1 Expression by WB Analysis

**Figure 5:**
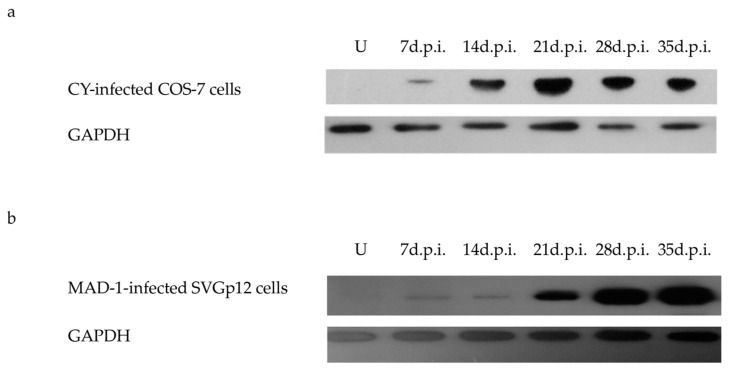
WB analysis of the VP1 protein expressed in CY- and MAD-1-infected cells after infection. Cell protein extracts from uninfected (U) and infected COS-7 and SVGp12 cells were harvested at 7 d.p.i., 14 d.p.i., 21 d.p.i., 28 d.p.i., and 35 d.p.i., and analyzed by WB to evaluate the expression of the VP1 protein. Equal amounts of protein from whole-cell protein extracts were separated by SDS–PAGE, transferred to PVDF, and probed using anti-VP1 antibody. Normalization to the host cell number (VP1/GAPDH) was performed. (**a**) Results showing that the VP1 protein in the CY-infected COS-7 cells was expressed 7 d.p.i., and its level increased during the infection experiments, reaching the maximum expression at 21 d.p.i., although remaining detectable for up to 35 d.p.i. (**b**) Results showing that the VP1 protein in the MAD-1-infected SVGp12 cells was expressed 7 d.p.i., and its level increased during the infection experiments, reaching the maximum expression at 35 d.p.i.

To confirm that the expression of the JCPyV late genes occurred in freshly seeded COS-7 and SVGp12 cell lines infected with supernatants containing a known amount of virions obtained from previous transfection experiments, WB was performed on VP1. Interestingly, the results showed that the VP1 protein in the CY-infected COS-7 cells was detected as early as 7 d.p.i., and its levels increased during the infection experiments, reaching the maximum expression at 21 d.p.i. (Figure 5a). The amount of VP1 expression was greater in SVGp12 cells infected with the MAD-1 strain (Figure 5b) than in the CY-infected COS-7 cells and in the MAD-1-infected COS-7 cells.

## 4. Discussion

JCPyV is characterized by establishing persistent infections in healthy hosts and generally causing clinical disease only in hosts whose immune systems are compromised [51]. Even though JCPyV was discovered five decades ago [52], the knowledge of the mechanisms that govern its viral persistence and reactivation is limited [51,53]. In this context, the activation of JCPyV replication in different cell compartments is associated with the presence of several host transcription factors [54,55,56]. Moreover, JCPyV replication in brain tissue is dependent on the rearrangements of the NCCR of the viral genome, which increase the number of binding sites for cellular transcription factors [54,55,56]. The ability of a viral genome to persist without destroying the host cell affords the virus a means of avoiding recognition by the immune response and clearance of the virus from the organism. Thus, in a persistent state, a virus minimizes synthesis of viral antigens that might alert the host to the infection [51]. It has been proposed that the regulation of JCPyV’s persistence and reactivation might depend not only on immune or epigenetic regulation, but also on viral miRNAs. As mentioned above, JCPyV encodes for miRNAs that are complementary to early transcripts and, therefore, have the potential to negatively regulate the EVGR expression and are likely involved in the regulation of viral replication [30,57]. So far, two major mechanisms of regulating JCPyV replication have emerged, involving a transcriptional, NCCR-based structure and a post-transcriptional miRNA-based mechanism targeting EVGR expression. Moreover, JCPyV miRNA targets the mRNA of the ULBP3 protein, which is the ligand of natural killer cell receptor NKG2D. Consequently, JCPyV-infected cells can avoid being destroyed by NK cells, enabling the virus to establish a persistent infection [58].

In this study, the interplay between JCPyV replication based on the NCCR’s structural architecture and the miRNA levels following JCPyV infection with archetype CY and the *rr*-NCCR-JCPyV variant MAD-1 was explored, following JCPyV infection in two different cell lines to represent different sites of viral persistence.

Specifically, to pursue this goal, the viral replication of the CY variant of JCPyV possessing the archetypal non-pathogenic NCCR structure, along with the MAD-1 isolate showing the *rr*-pathogenic NCCR structure, was investigated in COS-7 and SVGp12 cells.

Following infection, JCPyV loads rapidly increased in MAD-1-infected cells, with similar kinetics in both cell lines, but only slowly in archetype-CY-infected cultures, in which faster replication kinetics were observed in COS-7 cells than SVGp12 cells. As shown previously in COS-7 cells [59,60], and, for the first time for SVGp12, the viral replication cycle was complete for both strains, and a secondary infection event had started, as evidenced by the LT Ag-positive replication and VP1-positive WB assay.

Interestingly, WB showed VP1 protein expression as early as 7 d.p.i., and its levels increased during the infection experiments, reaching the maximum expression at 21 d.p.i.

The levels of the VP1 protein were higher in SVGp12 cells infected with the MAD-1 strain compared with the CY-infected COS-7 and SVGp12 cells and the MAD-infected COS-7 cells. This is likely the result of increased replication capacity, because it is well known that polyomavirus genome amplification results in increased late gene expression [61,62].

Our results confirm that COS-7 and SVGp12 cells can be used to obtain large-scale production of JCPyV. and show that these systems can be used to better understand the biological bases of JCPyV and its mechanisms of infection, potentially opening new roads to the control and blockage of viral transmission and reactivation. To the best of our knowledge, although the miR-J1-5p expression in COS-7 was previously shown at different times [63], this is the first description of an in vitro model allowing the study of the production and the kinetics of miR-J1-5p for 35 d.p.i., using not only the archetypal CY strain but also the *rr*-MAD-1 strain. In addition, the SVGp12 cell line was also used to evaluate the miR-J1-5p expression after JCPyV infection with both strains.

Interestingly, the miR-J1-5p expression was inversely correlated with the JCPyV replication trend. In detail, low JCPyV miRNA-5p levels were observed in infection by rapidly replicating JCPyV-carrying *rr*-NCCRs, whereas high miRNA-5p levels were measured in the CY JCPyV strain, in which low levels of viral replication were observed in both cell lines.

Although both cell lines used in this study express the SV40 large T antigen, it is unlikely that JCPyV miR-J1-5p targets the SV40 large T antigen mRNA, because there is only 50% identity between miR-J1-5p and SV40 microRNA [29,30]. Moreover, JCPyV does not replicate in parental COS-7 cells, which do not express the SV40 T antigen [64], whereas JCPyV replication was observed in COS-7 and SVG12p cells despite the expression of JCPyV miR-J1-5p.

The exact molecular mechanisms need to be addressed further, but our results provide an account of the inverse association of EVGR activity (LTAg) and miRNA-5p expression. In this scenario, it is possible to speculate that the expression of viral miRNAs may be involved in the control of JCPyV replication, which is required for asymptomatic viral persistence. The JCPyV miRNA-5p has been proposed to act as an important safeguard, silencing residual LTAg expression during viral persistence. Conversely, the reduction in JCPyV-miRNA expression can be associated with viral reactivation or increased replication.

In this study, the expression of viral miRNAs was also investigated in exosomes, because increasing evidence has shown that exosomes may be exploited by viruses such as HIV, HSV, and EBV, to provide additional routes for the transmission of viral infections, protecting them from immune recognition, exhibiting pro-inflammatory and anti-inflammatory effects on cells, and thereby facilitating persistent infections in the host [65]. Similar to these viruses, it is hypothesized that during JCPyV infection, the exosomes containing viral miRNAs delivered to uninfected cells could be a potential viral counteracting mechanism involved in the repression of viral replication to maintain JCPyV asymptomatically in the host.

For these reasons, firstly, we investigated whether the JCPyV miRNAs were expressed in the exosomes produced by the COS-7 and SVGp12 cell-free supernatants. The results showed that JCPyV miRNA was present in the exosomes starting from 14 d.p.i. and reached the peak of expression at 21d.p.i. A significantly higher expression of miRNA levels was observed in exosomes generated during archetypal JCPyV infection than was observed for the *rr*-NCCR. It is possible that the lower levels of JCPyV miRNA in exosomes generated during archetypal JCPyV infection—compared to those present in exosomes generated during infection with the *rr*-JCPyV strain—were due to a reduction in the production of exosomes during high viral replication, or to the cellular uptake of the exosomes’ viral miRNAs present in the cell-free supernatant.

To further confirm that the JCPyV replication could regulate the viral miRNA expression, for the first time, the replication of archetypal and MAD-1 JCPyV strains and viral miRNA expression in the exosomes present in COS-7 and SVGp12 cell supernatants were concomitantly investigated at an early time point after infection (24 h p.i.). Our results confirmed the presence of replication and VP1 expression in COS-7 and SVGp12 cells infected with the MAD-1 strain, along with the lack of viral replication and VP1 production in both cell lines infected with the archetypal JC viral strain. JCPyV miRNA expression was consistently detected in well-characterized exosomes present in high concentrations in supernatants obtained from CY-infected cells. These results are consistent with those obtained by Giovannelli et al. [63], who found that JCPyV miRNAs in exosomes were present from 12 h post-infection and, using the JCPyV archetype, that miRNA expression was present early on, unlike in JCPyV with *rr*-NCCR. The possibility that JCPyV miRNAs were able to downregulate the replication of JCPyV variants was refuted in the course of the experiments by adding JCPyV-miRNA-5p-loaded exosome preparations to cells prior to infection. The latter event could be exploited to support the fact that JCPyV-miRNAs in the exosomes could induce a latent/persistent viral state in cell cultures, underlining the potential role of exosomes and miRNA cargo in cell–cell communication, and offering an interesting possibility that could potentially be harnessed for antiviral therapy.

## 5. Conclusions

In conclusion, this study reiterates that the nature of the polyomavirus NCCR plays a key role in viral replication. In particular, in JCPyV carrying an archetypal NCCR form and possessing promoter elements producing high late gene expression, the high expressions of miRNAs inhibit LTAg detection and, consequently, allow low viral DNA replication and persistence in both cell lines considered to be sites of viral persistence. Conversely, for JCPyV carrying an rr-NCCR form, in which viral early gene expression is increased by altered promoter elements, the low miRNA expression does not inhibit LTAg detection, thereby inducing high-level viral replication, with all of its potential pathological consequences. It should be noted that single mutations in the NCCR transcription binding sites of the promoter elements playing a role at the start of the switch from late promoter activity to early promoter activity may overcome the repression of miRNA expression during viral replication. Additional investigations of the expression of JCPyV miRNAs, and of their presence in exosomes, are required to shed light on their regulatory role during viral reactivation.

## Figures and Tables

**Table 1:** Differences in viral replication with and without exosomes as a percentage of inhibition.

**CY in COS-7**						
	**3**	**7**	**14**	**21**	**28**	**35**
**Without exosomes**	2 × 10^3^	9 × 10^3^	10^4^	7 × 10^4^	5 × 10^5^	8 × 10^5^
**With exosomes**	8 × 10^2^	10^3^	5 × 10^3^	8 × 10^3^	4 × 10^4^	9 × 10^4^
**% inhibition**	60%	89%	50%	89%	82%	89%
**MAD-1 in COS-7**						
	**3**	**7**	**14**	**21**	**28**	**35**
**Without exosomes**	10^4^	8.5 × 10^4^	6 × 10^5^	9 × 10^6^	7 × 10^7^	9 × 10^8^
**With exosomes**	7 × 10^2^	9 × 10^2^	3 × 10^3^	6 × 10^3^	10^4^	5 × 10^4^
**% inhibition**	93%	99%	99.5%	99.9%	>99.9%	>99.9%
**CY in SVG12p**						
	**3**	**7**	**14**	**21**	**28**	**35**
**Without exosomes**	9 × 10^2^	2 × 10^3^	9 × 10^3^	5 × 10^4^	10^5^	5 × 10^5^
**With exosomes**	10^2^	7.5 × 10^2^	10^3^	9.5 × 10^3^	7 × 10^4^	10^5^
**% inhibition**	89%	63%	89%	81%	30%	80%
**MAD-1 in SVG12p**						
	**3**	**7**	**14**	**21**	**28**	**35**
**Without exosomes**	9 × 10^4^	3 × 10^5^	7.5 × 10^5^	6 × 10^6^	8.5 × 10^7^	8.5 × 10^8^
**With exosomes**	10^2^	5.5 × 10^2^	9 × 10^2^	5.5 × 10^3^	5 × 10^3^	9 × 10^4^
**% inhibition**	99.9%	99.8%	99.9%	99.9%	>99.9%	>99.9%

## Data Availability

Data are contained within the article or the Supplementary Materials.

## Supplementary Materials

The following supporting information can be downloaded at: www.mdpi.com/article/10.3390/v14092070/s1, Table S1: JCPyV replication at different time point in the CY and MAD-1-infected COS-7 and SVGp12 cell lines; Table S1bis (based on data from Table S1). Fold-increase of the JCPyV copy number starting three days after infection in the CY and MAD-1-infected COS-7 and SVGp12 cell lines; Table S2: miR-J1-5p expression at different time point in the CY and MAD-1-infected COS-7 and SVGp12 cell lines; Table S2bis (based on data from Table S2): Percent decrease (%-age decrease) of miR-J1-5p expression at different time point in the CY and MAD-1-infected COS-7 and SVGp12 cell lines; Table S3: miR-J1-5p expression at different time point in the exosomes after COS-7 and SVGp12 cells-infection with CY and MAD-1 strains; Table S3bis (based on data from Table S3): Percent decrease (%-age decrease) of miR-J1-5p expression at different time point in the exosomes after COS-7 and SVGp12 cells-infection with CY and MAD-1 strains; Table S4: JCPyV replication post-infection addition of JCPyV miRNA-containing exosomes to uninfected cell lines COS-7 and SVGp12 cell lines; Table S4bis (based on data from Table S4): Fold increase of JCPyV replication post-infection addition of JCPyV miRNA-containing exosomes to uninfected cell lines COS-7 and SVGp12 cell lines.
