# Supplementary material for: COS-7 and SVGp12 Cellular Models to Study JCPyV Replication and MicroRNA Expression after Infection with Archetypal and Rearranged-NCCR Viral Strains
Source: Viruses. 2022 Sep 17;14(9):2070. doi: 10.3390/v14092070 (PMC9502812; doi:10.3390/v14092070)
Supplement: Supplementary file 1 [file viruses-14-02070-s001.zip › viruses-1894352-supplementary.pdf]

Table S1. JCPyV replication at different time points in the CY and MAD-1-infected COS-7 and SVGp12 cell lines

archetype CY-infected cells	JCPyV average value copies-cell/days post-infection (d.p.i.)					
	**3**	**7**	**14**	**21**	**28**	**35**
COS-7	2,00E+03	9,00E+03	1,00E+04	7,00E+04	5,00E+05	8,00E+05
SVGp12	9,00E+02	2,00E+03	9,00E+03	5,00E+04	1,00E+05	5,00E+05
**MAD-1-infected cells**	**JCPyV average value copies-cell/ days post-infection (d.p.i.)**					
	**3**	**7**	**14**	**21**	**28**	**35**
COS-7	1,00E+04	8,50E+04	6,00E+05	9,00E+06	7,00E+07	9,00E+08
SVGp12	9,00E+04	3,00E+05	7,50E+05	6,00E+06	8,50E+07	8,50E+08

Table S1bis (based on data from Table S1). Fold-increase of the JCPyV copy number starting three days after infection in the CY and MAD-1-infected COS-7 and SVGp12 cell lines

			CY strain			
	3	7	14	21	28	35
COS cells	set as 1	4.5-fold	5-fold	35-fold	250-fold	400-fold
SVG12p	set as 1	2.2-fold	10-fold	56-fold	111-fold	556-fold
			**MAD-1 strain**			
COS cells	set as 1	8.5-fold	60-fold	900-fold	7,000-fold	90,000-fold
SVG12p	set as 1	3.3-fold	8.3-fold	67-fold	944-fold	9,444-fold

Table S2. miR-J1-5p expression at different time points in the CY and MAD-1-infected COS-7 and SVGp12 cell lines

archetype CY-infected cells	JCPyV average value (copies-ng)/days post-infection (d.p.i.)					
	3	7	14	21	28	35
COS-7	900	800	700	650	600	540
SVGp12	950	900	850	700	650	600
**MAD-1-infected cells**	**JCPyV average value (copies-ng)/days post-infection (d.p.i.)**					
	3	7	14	21	28	35
COS-7	850	780	550	500	350	250
SVGp12	800	550	500	400	320	200

Table S2bis (based on data from Table S2). Percent decrease (%-age decrease) of miR-J1-5p expression at different time points in the CY and MAD-1-infected COS-7 and SVGp12 cell lines

CY strain						
	3	7	14	21	28	35
COS-7	set as 100%	11%	22%	28%	33%	40%
SVG12p	set as 100%	5%	11%	26%	32%	37%
**MAD-1 strain**						
COS cells	set as 100%	8%	35%	41%	59%	71%
SVG12p	set as 100%	31%	37%	50%	60%	75%

Table S3. miR-J1-5p expression at different time points in the exosomes after COS-7 and SVGp12 cells-infection with CY and MAD-1 strains

archetype CY-infected cells	JCPyV average value copies-ng/days post-infection (d.p.i.)					
	3	7	14	21	28	35
COS-7	500	500	400	300	300	250
SVGp12	550	500	400	350	300	300
**MAD-1-infected cells**	**JCPyV average value copies-ng/days post-infection (d.p.i.)**					
	3	7	14	21	28	35
COS-7	350	350	300	300	250	200
SVGp12	300	300	280	250	230	150

Table S3bis (based on data from Table 3): Percent decrease (%-age decrease) of miR-J1-5p expression at different time points in the exosomes after COS-7 and SVGp12 cells-infection with CY and MAD-1 strains

CY strain						
	3	7	14	21	28	35
COS-7	set as 100%	0%	20%	40%	40%	50%
SVG12p	set as 100%	9%	27%	36%	45%	45%
**MAD-1 strain**						
COS cells	set as 100%	0%	14%	14%	29%	43%
SVG12p	set as 100%	0%	7%	17%	23%	50%

Table S4. JCPyV replication post-infection addition of JCPyV miRNA-containing exosomes to uninfected cell lines COS-7 and SVGp12 cell lines

**archetype CY-infected cells**	**JCPyV average value (copies-cell)/ days post-infection (d.p.i.)**					
	**3**	**7**	**14**	**21**	**28**	**35**
COS-7	8,00E+02	1,00E+03	5,00E+03	8,00E+03	4,00E+04	9,00E+04
SVGp12	1,00E+02	7,50E+02	1,00E+03	9,50E+03	7,00E+04	1,00E+05

**MAD-1-infected cells**	**JCPyV average value (copies-cell)/ days post-infection (d.p.i.)**					
	**3**	**7**	**14**	**21**	**28**	**35**
COS-7	7,00E+02	9,00E+02	3,00E+03	6,00E+03	1,00E+04	5,00E+04
SVGp12	1,00E+02	5,50E+02	9,00E+02	5,50E+03	5,00E+03	9,00E+04

Table S4bis (based on data from Table 4). Fold increase of JCPyV replication post-infection addition of JCPyV miRNA-containing exosomes to uninfected cell lines COS-7 and SVGp12 cell lines

	**CY strain**					
	3	7	14	21	28	35
COS-7	set as 1	1.3-fold	6.3-fold	10-fold	50-fold	113-fold
SVG12p	set as 1	7.5-fold	10-fold	95-fold	700-fold	1000-fold
	**MAD-1 strain**					
COS cells	set as 1	1.3-fold	4.3-fold	8.6-fold	14-fold	71-fold
SVG12p	set as 1	5.5-fold	9-fold	55-fold	50-fold	900-fold
